# “Never Again the Everyday”: On Cinema, Colportage and the Pedagogical Possibilities of Escapism

**DOI:** 10.1007/s11217-021-09781-w

**Published:** 2021-07-18

**Authors:** Marie Hållander

**Affiliations:** Department of Culture and Education, Södertörns Högskola, Stockholm, Alfred Nobels Allé 7, Flemingsberg, 141 89 Huddinge, Sweden

**Keywords:** Ernst bloch, Walter benjamin, Escapism, Daydreaming, Hope, Utopia, TV series, Distraction, Pedagogical possibilities

## Abstract

This article is a philosophical analysis of escapism as a pedagogical possibility, with a particular focus on TV series. Taking my own, as well as students, experience of escapism into TV series as a starting point, that is, their ability take us somewhere far away, something which has become more acute during the pandemic time since we remain more or less self-isolated because of the corona virus Covid-19, the article discusses escapism in relation to distraction and attention in life as well as within teaching, but also in relation to colportage, hope and social justice. According to Ernst Bloch, social justice cannot materialize without regarding things differently. Something that is often dismissed as mere escapism might be a seed for a new and more humane social order, as it can be seen as an “immature, but honest substitute for revolution” (Bloch 1986, p. 368). Drawing on Bloch’s understanding of colportage and hope, as well as Walter Benjamin’s understanding of mass culture and cinema, the article treats escapism and TV series not as something simple, but rather as possible seeds for a new social order and thus as having pedagogical possibilities (Hållander, 2020).

## Introduction

“I move. From early on we are searching. All we do is crave, cry out. Do not have what we want.” (Bloch 1986, p. 21)

I am sitting with the students, we are in a circle, in the classroom. We are about to begin, and I sigh and hum to get the students’ attention. I start speaking, say hello, introduce today’s lecture and so on. Most of the students are looking at me, taking notes, but two students are not, they are looking at their phones in front them, headphones in their ears. I suppose they are watching something; they are not focusing on me, they are concentrating on something else. They seem be to escaping from this room, the classroom, to another room, nearby but at the same time distant. They are here, and at the same time, not.

On another occasion, I see the same thing happen, and this time I see what one of the students is watching: an American sitcom.

I feel her. I, like her, like sitcoms. I have consumed a great deal of TV series myself. Since early childhood I have been sitting in front of the TV, eating my dinner, dreaming away to other worlds. I remember longing for the telly, more so than now, when I was young. There I did not have to perform, or act or be *someone,* like in school. I could just … be myself, or rather, I could be *somewhere* else. Not here, but far away. But still in the present. I followed *Anne of Green Gables* and her struggle for a family, and later friends and love. I took part in the friends’ struggle in *Beverly Hills* 90210, as well as those of the six *Friends* in New York during the 1990s. This has followed me into the adult world and through today’s access to series like *SKAM* and *Lovecraft Country* distributed through channels like Netflix, HBO and SVT (Swedish Television). The series are always there, accessible.

Television and the culture of series on streaming sites like Netflix or HBO are a part of the art of motion-picture photography, that is cinema. At the time of writing, the world is in the midst of a pandemic, where it is, in one sense, our duty as citizens to stay on the sofa as we remain more or less self-isolated because of the corona virus Covid-19. This has also shaped the phenomenon of cinema, since we cannot any longer visit theatres to watch movies but are referred to our sofas. This heightens the importance and relevance, I argue, of trying to understand what this phenomenon (of escapism into) TV series means philosophically and pedagogically. To analyse the significance of these emergent trends in digital media use, the article also draws on biographical elements of my own binge watching and escapism through TV series.

Watching TV series is huge part of (youth) culture, referred to among other things as ‘the Netflix-effect’ (Matrix 2014). As Matrix (2014) states this refers to the intense consumption of TV series, in the form of *binge watching* (watching a whole season of a series during a very short time) and discussing it on social media afterwards.^1^ Sharing thoughts and reviews, discussing characters and the plot are a part of a widespread culture. This practise does not stay outside; it enters the classroom, hence students not paying attention to the teacher or studying but watching TV series or playing games on their phones or iPad instead (cf. Rytzler 2017), as my earlier example also showed, i.e. the students were escaping from what they are supposed to do.

Escapism is defined in the *Cambridge English Dictionary* as “a way of avoiding an unpleasant or boring life, especially by thinking, reading, etc. about more exciting but impossible activities.” This definition regards escapism in the form of both daydreaming and avoiding (thinking about something) and as escaping into something, for example, a TV series.^2^ In this article I want to stay with this definition, but also develop this phenomenon of escapism, especially into TV series, pedagogically. I do this by asking: What are the pedagogical possibilities of escapism and TV series?

In what follows I will relate the question of the pedagogical possibilities to the subjects’ transformation in terms of empowerment and freedom (ref. Blinded manuscript) as well as to formal education and teaching, which will be made clear throughout the article. In my book (Hållander, 2020) I discuss the pedagogical possibility, (specifically in relation to the phenomenon of testimony and witnessing) in relation to Agamben’s understanding of potentiality. Among others I regard it in relation to time, as a possibility that is *not yet*, but *can* be. I write: “This means that the pedagogical possibilities for the child at school are not placed in the future, in adult life, but rather in the here and now, and that this possibility does not include a determination or a direction, nor does it always enter into a realisation.” (Hållander, 2020, p. 28) Pedagogical possibilities I relate to politics, ethics, aesthetics and the societal, and as moments of transformations where existential and emancipatory becoming can take place. (Hållander, 2020, p. 22).

With a particular focus on TV series in this article, I want to offer a nuanced picture of escapism as not just something problematic within teaching. Instead, I attempt in to argue that escapism could be the seed of the very beginning of change, a form of transformation and, therefore, a phenomenon relevant to pedagogy. To do this, the article will be developed into three different directions by, first, discussing the problem of distraction, attention, mass culture and movies, mainly drawing on Walter Benjamin, second, discussing attention formation within teaching, and third, discussing Ernst Bloch and the relations between the fairy tale, colportage and hope in relation to action and social justice. Through these three developments, the article formulates an argument that escapism is not simply the opposite of studying but rather can be formulated as slowing things down, as creating space for reflection and rest, and ultimately, as an immature, yet honest, beginning of change and therefore as having pedagogical possibilities (Hållander, 2020).

## TV Series, a Culture for the Distracted Masses: A Political Failure?

As the introduction of this special issue states: “Cinema is definitely a mass art—reaching out to millions of teens—by not only a broad distribution and easy access, but also because it speaks a language youths can relate to” (Schumann and Strand 2021, forthcoming). TV series are, of course, also a part of mass culture; this is not something new. Walter Benjamin states in the essay “The Work of Art in the Age of Mechanical Reproduction”, in a discussion on movies and reproduction of art, how: “Mechanical reproduction of art changes the reaction of masses towards art” (Benjamin 1969, p. 234). The masses, the people, are distracted by as well as fixated on and drawn into the art form of movies. This statement also works today. Binge watching Netflix series and scrolling videos on Facebook, Instagram or YouTube have changed people’s reactions toward images and art, and have become phenomena that are a part of a mass culture.

At the same time, as Benjamin also discusses, the masses are mistrusted (Benjamin 1969). Drawing on Duhamel, he discusses how the movie has been the cultural form for the masses, for the uneducated, and the proletariat: a culture for the masses that creates ideas and hopes for the future, “which kindles no light in the heart and awakens no hope other than the ridiculous one of someday becoming a ‘star’ in Los Angeles” (Benjamin 1969, p. 239). This phenomenon (of being a star in Los Angeles) is at work here in the Nordic Countries even though Hollywood and Los Angeles are faraway places that few from here even visit. The phenomenon works as it continues to be portrayed through TV series and cinema. The dream of pearls, diamonds, fame and luxury is of course nothing new; have humans not always had that dream? This culture is referred to by Benjamin as a “spectacle which requires no concentration and presupposes no intelligence” (Benjamin 1969, p. 239). To escape into TV series demands nothing of the spectator. Benjamin ends the essay with the following:

The film with its shock affects meets this mode of reception halfway. The film makes the cult value recede into the background not only by putting the public in the position of the critic, but also by the fact that at the movies this position requires no attention. The public is an examiner, but an absent-minded one. (Benjamin 1969, pp. 240–241)

You can be a part of mass culture, watch movies, without having to do anything, seems to be the conclusion. The public are the examiners, but absent-minded ones; they are dreaming. It is as if they were escaping from the reality or the facts of the world. The distracted masses, the growing proletariat and working classes, becomes a problem, leads to passivity and, in the end, always, he writes, war (Benjamin 1969, p. 241). Translating this argument of the distracted masses into escapism and TV series; escapism would be regarded as a failure, in a political sense. Escapism does not lead to action. Rather, it makes us passive, as Benjamin mentioned.

Staying with the relation between action, escapism and class, an interesting analysis of escapism in relation to class comes from Steph Lawler and her reading of the working-class women in the novel *Breezeblock Park* by Valerie Walkerdine (Lawler 2000). Lawler points outs the importance of differentiating between *escape* and *escapism.* In her reading of the representation of working class women she notices the contrast between the daughter’s escape and the mother’s escapism. When working class kids escape from their background it is regarded as a success, whereas escapism, when doing the dishes or watching TV and dreaming of that *other world*, is only to be regarded as a failure. An incomplete escape. Lawler problematizes this conclusion by ending her analysis by stating that it is also to frame working class problems and dreams as the counter-part to those of the middle class, and thus poses the question, “Is this why escape is heroic, while escapism can only be regarded as failure?” Escapism can be regarded as a failure, but through which lens? Whose escape, and from where? As I want to argue in this article, and later on related to Bloch’s view that escapism is something honest, even though it is immature, I would rather argue that one cannot speak of escapism as something incomplete or as a failure, but rather as a point of departure. As a beginning.

Also, Benjamin, who wrote the text as a Marxist analysis of art in the age of capitalist production and Fascism, states that his theses have nothing useful for Fascism but, on the other hand, are “useful for the formulation of revolutionary demands in the politics of art” (Benjamin 1969, p. 218). The text is therefor to be read as a defender of the revolutionary possibility of art in modern times.

Benjamin’s text on art in the age of mechanical reproduction (Benjamin 1969) leads me to this article’s question of what the pedagogical possibilities of escapism in relation to TV series can be understood as, and more specifically here, to the question of the function of distraction and attention. To study one needs to focus, to be attentive. But escapism is often perceived as being distracted. Benjamin asks for a closer look at the difference between concentration and distraction and cinema and writes that: “Distraction and concentration form polar opposites which may be stated as follows: A man who concentrates before a work of art is absorbed by it. (…) In contrast, the distracted mass absorbs the work of art” (Benjamin 1969, p. 239). Here Benjamin is also referring to the masses, the growing proletariat, and how they are, first and foremost, distracted, but also, second, how they absorb the work of art. This quotation is interesting in relation to two different aspects, its reference to the masses, the proletariat and the working classes and their use of culture, and its reference to the phenomenon of distraction. I will return later to the question of culture and class, in the context of a discussion on the pedagogical possibility and, drawing on Bloch, revolutionary possibility of escapism. Next, I will continue to consider distraction and attention.

## Escapism as Distraction or Attention? A Question for Education

What Benjamin is proposing is that the act of watching movies makes one unable to give the attention needed. In comparison to paintings, he writes:

The painting invites the spectator to contemplation; before it the spectator can abandon himself to contemplation. Before the movie he cannot do so. No sooner has his eye grasped a scene than it is already changed. It cannot be arrested. (Benjamin 1969, p. 238)

The rapidity of movie scenes and quantity of video clips have increased exponentially since Benjamin wrote this. I could write the same of the experience of watching *vines* (very short video clips) on YouTube with my daughter; as soon as I have grasped one scene it has already changed. I cannot comprehend it (even though my daughter seems to).

However, and in contrast to Walter Benjamin, to grasp the scenes that are being shown, one does need to concentrate. One cannot be distracted, one needs to be attentive. Concentration and distraction—how do they feature in relation to watching TV series? The line between distraction and concentration is perhaps not so sharp; they are perhaps not such opposing phenomena as Benjamin suggests. Perhaps one could regard them as more interrelated? In the following I will discuss how these phenomena—attention, concentration and distraction—are interrelated.

If we regard studying as a central part of teaching (Masschelein and Simons 2013; Lewis 2013), then escapism can be, or is, regarded as a problem. If students are daydreaming instead of studying, they are not taking part in what is to be studied. They are not *there*. How should one regard this being there, but also not being there, within teaching? As forms of resistance? As a form of slowing things down? It could be regarded as a form of a resistance, as, consciously or unconsciously, a way of not joining the common. Escapism tends to be regarded as a form of distraction; if students fly away to another/parallel reality than the classroom, they are regarded as distracted. They are not paying attention. They are not studying.

However, as Rytzler (2017) has discussed in *Teaching as attention formation*, attention is central for thinking of education, teaching, as well as what it means to study. As Rytzler states: “The question of attention is easily turned into a question of distraction” (2017, p. 56). Even so, Rytzler continues: “[w]hen schools deal with the problem of distraction, (…) they often do it within the frames of a new biomedical governance that has come to influence and to some extent even characterize the educational system” (Rytzler 2017, p. 56). This biomedical governance within education makes a clear distinction between what is normal attention formation and what is not; for example, when students need medical treatment for studying. Rytzler draws on Rancière and continues to write how “[d]istraction is not the inability to pay attention but rather the refusal to acknowledge the fundamental inequality that, to Rancière, so often characterizes educational relations” (Rytzler 2017, p. 109). Rytzler continues: “This way of looking at education becomes possible if one regards the distinction between attention and distraction as one of politics rather than logic” (Rytzler 2017, pp. 109–110). In this way Rytzler problematizes what attention, as well as distraction, can mean within education, and how it is constructed as partly a formation of inequality as well as politics, rather than logic, evident in the way it has also become a bio-medical problem within education. Rytzler argues how attention formation becomes the focus for education—as well as a didactic question of *how* teachers can get the students’ attention through teaching methods.

As Rytzler points out, the line between attention and distraction is not as clear as we might think. Students are not either attentive or distracted: when students talk or listen to another student, or are absorbed into the world of a TV series on their phones, they are still attentive. The act of escapism within education can, through this lens of attention/distraction, also be formulated as a political question rather than a logical question. Escapism or dreaming in classrooms is attentive—but attentive to the wrong things, which becomes a problem within teaching.

To sum up, I have developed notions of culture for the masses and escapism within teaching in relation to attention and distraction and put forward that, although escapism can be considered to be undesirable within teaching (as the students are not paying attention to the right things) it can be regarded as a form of concentration and attention (and not their opposite). In the following I will, by turning to Bloch, discuss escapism in terms of liberty and freedom (which become something different than we find in a neo-liberal education). To this end, the article will, with the help of Bloch and Benjamin, develop an understanding of escapism as a pedagogical phenomenon that could be the very start to changing things (in Bloch’s terms, a beginning of a revolution). I will therefore leave the classroom for now and speak about the pedagogical possibilities of escapism in terms of transformation, empowerment and freedom, by beginning with Ernst Bloch’s understanding of escapism in relation to colportage, fairy tale and hope. I will come back to the classroom later on in the conclusion.

## The Fairy Tales of Netflix: As Colportage?

I follow the fairy tales of the heroes from my sofa^3^. And just as Benjamin is writing, I can see how this viewing, this escapism into the TV series, does not require anything of me. Even though I am attentive, escapism is pleasant, requires no intelligence or action. During the pandemic of Covid-19, I have seen Atticus Freeman and Letitia fight monsters and racism in *Lovecraft Country* on Netflix. The program is about a young black man who travels across the segregated United States during the 1950s in search of his father and encounters all sorts of dark secrets, which plague the city. The show is inspired by the famous horror writer H.P. Lovecraft's fictional stories. But my, and others', viewing of this program has hardly led to anything more than a moment's relaxation. On the couch I can dream away from my own world into another reality and let Atticus and Letitia—the heroes in *Lovecraft Country*—do the work that I myself do not have the energy to. As stated before, escapism is therefore to be regarded as a failure, in a political sense. It does not lead to action. Rather, it makes us passive, as Benjamin mentioned.

Ernst Bloch, the Jewish Marxist philosopher, offers further perspectives on escapism and—not TV series, of course, since it is after his time—but the forerunner, of colportage literature in his work *The Principle of Hope*, which was published in three volumes in 1954, 1955, and 1959. Bloch writes in volume one that the: “The sweeping fairy tale is the adventure story, it best lives on today as *colportage*” (Bloch 1986, p. 367). Colportage and colportage literature was a special form of literature, filled with intrigue, violence and drama and has been given the status of "inferior literature" or as “bad novels” for its simple personal descriptions and deficient language (Hedman 2017; *Svenska akademiens ordlista över svenska språket* 2006). In order to make this idea of colportage understandable and why it is relevant in relation to the pedagogical possibility of escapism, I will make a detour around these forms of novels, as well as function of giving hope to the people, and then come back to Bloch’s understanding.

The word *Colportage* refers to the way how these novels, and books and religious tracts where distributed, by carriers on horses called “colporteurs” or “colporters” during the nineteenth century. The word *col* is derived from the Latin *collum*, "neck", and thus refers to the horse's neck; the horse that the colporteurs rode in the rural area where they then knocked on the doors and sold the books.

That the colportage literature was considered as inferior came only later, writes Dag Hedman in an essay on the early Swedish crime story (Hedman 2017). In the early nineteenth century, it was simply in the way that novels could be printed, as a periodic division of publications of larger works, sheet by sheet. The distribution consisted of small booklets, the scope consisted of 16, or 24 and 36 pages that were distributed every week for a cheap price (often 10 öre each). The authors of the colporter literature thus had their novels printed for a long time, sometimes several years, and in order to keep interest up and lead to a continued subscription, the novels contained strong intrigue and were real page turners (Hedman 2017). Through this way of distributing and printing literature, the poor and working class were given the opportunity, through the low price and the long-term subscription, to obtain extensive novels. Those who managed to keep up until the end of that publication could tear off the pages that did not include the novel and get a free publishing cover to bind the entire novel with (Hedman 2017).

Today we could see how the Netflix series have replaced this need for entertainment and stories filled with intrigue that colportage literature had before. The streaming series are full of intrigues, accessible and cheap with its monthly subscriptions that we can also share between different households and screens.

The colporters did not only spread serial novels, but they were also religious speakers/preachers who spread Christian writings. They appeared at a time when there were few opportunities to spread different messages. In Sweden the Conventical act (Konventikelplakatet) prevented other religious groups than the Church of Sweden from spreading their message. Therefore, it is conceivable that the colporters, the impermissible preachers within the free churches, spread constructive literature, moral teaching, together with Christian messages to the masses. A spread that also seemed educational: The purpose of the preachers was to give hope (despite all the toil and misery of earthly life, there will be a reward in heaven), and to improve the living conditions there and then.

After the end of the Conventicle Act in 1858, the colporteurs instead became the title of a traveling preacher. A colporter's letter was issued, giving the right to preach. These colporters preached, among other things, about abstinence from alcohol and extramarital sex—something we today perceive as problematic but was then a social revolution. The revival movement is one of those movements that, side by side with others (the sobriety, labor and women's movement) paved the way for a more democratic society in Sweden. Today we could see these colporters, both before and after the end of the Conventical act, as peddlers, as disseminators of revolutionary ideas that instilled hope and courage in the people.

Coming back to Ernst Bloch: colportage is a figure in his understanding of hope and social justice: Bloch writes: “The dream of colportage is: *never again the everyday*; and at the end stands: happiness, love, victory” (Bloch 1986, p. 367, my emphasis). The colportage carries the dreams, it tells the stories of a different world. Something that is not merely a fairy tale, but something more. According to the Marxist Bloch, social justice cannot materialize without regarding things differently. Something that is merely “daydreaming” or “escapism” might be a seed for a new and more humane social order. It can become the happiness, the love, and as he writes, victory. The colportage, and the fairy tales, become this; they, both the carrier and the stories, bring the freedom: “Here there is immature, but honest substitute for revolution, and where else did it express itself but in colportage?” (Bloch 1986, p. 368).

Notice that Bloch mentions escapism as both *immature* and as a *substitution*. It is not yet, it is not real, in that sense. It is a substitution. But it is, at the same time, *honest*. Here escapism in the form of fairy tales stands in relation to both hope and utopia (Bloch 1986). We cannot think of a different world if we have not imagined it. The fairy tales bring those different worlds to us.

The Colportage literature—or today, the series—can take us to different worlds, even when these are not completely perfect, according to Ernst Bloch: “Even in the fairy tale not everything immediately runs smoothly. There are giants and witches, they block off, make us spin all night, lead us astray” (Bloch 1986, p. 367). In the series, the heroes have their problems—it is shown not least in *Lovecraft Country* where Atticus and Letitia face all kinds of violence and mishaps. They die, and resurrect, meet fascists and racists next to giant monsters and enchanted girls. It is ambivalent, as Bloch writes:

It is ambivalent, can point to Ku Kluxers and fascists, even be a special stimulant for them; but the foul stench also in fact points to the cosy bourgeoisie’s justified mistrust of too much of the poor devil and his campfire. (Bloch 1986, p. 368)

The heroes of the Colporter literature do not wait for happiness to fall into their laps, rather the opposite. There is courage in the heroes who, like their readers, have nothing to lose. They ally with the poor, against control and power. This can also be said in relation to *Lovecraft Country*. The heroes of the series are led astray, spin, commit mischief, but there is a direction and resistance to racism and violence that cannot be misinterpreted. The heroes Atticus and Letita experience fear and are coping with terror, but despite the risk of losing both love and life, they rushes straight into the danger.

The point by Bloch is, that struggles in the stories—like in *Lovecraft Country*—do not make a difference to their capacity to make us dream away. Instead, they make us more intrigued: “There is a courage about the hero of colportage which, usually like its reader, has nothing to lose” (Bloch 1986, p. 367). Bloch follows:

Every adventure story breaks the moral commandment ‘‘Pray and work’; instead of the first cursing prevails, instead of the second the pirate ship appears, the rifleman not in the King’s pay. (Bloch 1986, p. 368)

Today, in late capitalist society, the moral commandment is perhaps not “pray and work”, but perhaps rather “consume and work”. The Netflix series, perhaps, can stand as a different movement in response to this commandment? Or am I hoping too much? I do not want to make a naïve parallel, first of all because Netflix series and binge watching do not cover one story but several. And second, it is not difficult to highlight the differences between the former pedlar on horseback, and the commercialised Netflix productions in terms of accessibility and distribution. Netflix is not driven by revolutionary ideas in the same way as the pedlars. One cannot say that a Netflix series is like colportage; but still, they do both carry fairy tales all the same. Third, it could also easily be argued how the series make us more passive, or how the Netflix series are too commercialized so that we are just drawn into the idea, their representation of “consume and work”, as Benjamin suggested when he wrote that mass culture “kindles no light in the heart and awakens no hope other than the ridiculous one of someday becoming a ‘star’ in Los Angeles” (Benjamin 1969, p. 239).

However, the colporter’s operated at a time when free meeting places were limited, and although the prevailing state of the pandemic differs in the digital age, we are also limited by the pandemic. The series can be an arena to go to, for *bildung* and for conversation. And, as I want to argue in this article by drawing on Bloch, consuming such mass culture can also be understood as a seed of a way dealing with the world and, therefore, also as a possibility. Remember Bloch’s words on how the fairy tale and the daydreaming, the colportage, can be immature, but nevertheless a real substitute for hope and revolution.

To understand how escapism is understood in Bloch’s magnum opus *The Principle of Hope* (Bloch 1986) one also needs to relate it to hope and utopian thinking. In the following I will therefore discuss the relation between escapism and utopia/hope in order to articulate the pedagogical possibility of escapism. First and foremost, Bloch argues, one must distinguish between different hopes, which I will now expand on.

## Concrete and Abstract Hope: On Action

“When someone dreams, they never remain rooted to the spot. They move almost at will away from the place or the state in which they find themselves.” (Bloch 1986, p. 24).

In contrast to escapism, hope and utopian thinking are well discussed within philosophy of education, from Freire’s (Freire and Freire 2004) work on hope, to more recent work on utopian thinking (Papastephanou 2016, 2018; Suissa 2001). As Papastephanou states:

A utopian urge manifests itself in all visions of life as it might be, from *escapist* and consumerist psychic discharge that satisfies ego-ideal dreaming, to Paradise, Golden Age or messianic narrativity, and down to projects of technological change and scientific advance.” (Papastephanou 2016, p. 33, my emphasis)

Utopian thinking can take different forms, from concrete consumerist ideals to Paradise and technological changes, but also it can take the form of escapism, as Papastephanou writes. As Cook (2018) points out, in the book *Imagine Futures: Hope, Risk and Uncertainty* Bloch specifically argues that while some kinds of utopian thinking are better than others, all kinds of utopian thinking are better than anti-utopian views that close off the future (Cook 2018, p. 25). Perhaps we could say the same thing for the act of escapism: some acts of escapism are better than others, but all acts of escapism are better than acts that close of the future. Bloch sees utopian thinking as an expression of hope and argues that, like hope, utopian thinking is about the unfinished nature of the world and, therefore, the uncertainty of the future (Cook 2018, p. 113). Considering our current conditions, of corona virus, as well as climate change, we cannot say otherwise than that the future is uncertain. Even to the extent that we cannot be sure that the coming generations will have a world to live in. And if they do, it will be a different world. However, for Bloch, thinking about the future, and utopian thinking, is not about a desire for a certain predestined future. Rather, Bloch argues that utopian thinking should be a catalyst for creating a better future (Bloch 1986).

Bloch differentiates between two types of utopian thinking, *concrete* and *abstract*, which can also help us distinguish between different kinds of escapism (Bloch 1986, p. 199). Bloch is highly critical of the content of utopian thinking. To make a difference between concrete and abstract utopias is therefore necessary, because what is possible in the future cannot be determined definitively in the present. Not all utopian thinking has the possibility to change things. Following Marx, Bloch writes: “no abstract ideals are realized, but rather the repressed elements of the new, humanized society, that is, of the concrete ideal, are set free” (Bloch 1986, p. 199). Bloch makes a distinction between abstract utopias that deal with wishful thinking and lack the will to change anything, and concrete utopias that reach forward to a real, possible future. By this Bloch is arguing that the former, abstract thinking, is a product of immature thoughts, and is born more of desire than hope (see Cook 2018, p. 113). Abstract utopian views are perhaps too blurry and foggy: too immature. Creating change from abstract utopias is in that sense impossible. However, as Cook writes:

For Bloch, concrete utopias bring hope together with action because although they are concerned with what ought to or should be, they nevertheless deal what is possible, and what can therefore be brought about—even in a miniscule way—by those who imagine them. (Cook 2018, p. 113)

Concrete utopian dreams differ in that they bring hope together with action. Concrete hopes are tangible and “real”, and open for action in a way that abstract utopias largely are not. This does not take away the possibilities of dreams or utopias that are unreachable: though they may not be possible to incorporate, may not lead to action (for example, compare with the understanding of action by Arendt 1998), they may still be, for want of a better word, *meaningful*.

To develop my argument for escapism as a pedagogical possibility it is important to differentiate between this aspect of concrete and abstract thinking. TV series can be regarded as something concrete one can escape into. To escape into the world of fiction is real: it is not an abstract utopian idea of freedom, or liberty, as a state, or a mind-set. This does not mean that the TV series are portraying a concrete utopia, but perhaps, as Bloch argued in relation to colportage and fairy tales, that TV series, the stories, could shape a concrete form, even if it is immature and naïve, of hope. Leaving Bloch and coming back to Benjamin, I would also like to relate this possibility of TV series to what Walter Benjamin wrote on art: “One of the foremost tasks of art has always been the creation of demand which could be fully satisfied only later” (Benjamin 1969, p. 237). Art creates something for the present, as well as for the future as it creates hopes, dreams and demands.

## Escapism as Action: An Arrested Time

In the following I want to take up the thread I started with Walter Benjamin: the idea that cinema is culture for the masses that absorbs the masses and makes them passive. I quote again from Benjamin: “In contrast, the distracted mass absorbs the work of art” (Benjamin 1969, p. 239). Here it is as if the masses become one with the art, that is, cinema: they cannot be separated. In his essay, he highlights the masses’ passivity. Mass culture creates no action and no reflection; one can consume mass culture without having to do anything. However, Benjamin is also contradictory in his statements. In the same essay, “The Work of Art in the Age of Mechanical Reproduction,” Benjamin concludes how the distracted persons, the proletariat, the masses, also form habits, and new tasks are able to be performed shaped by earlier experiences (called apperception in his text), and he concludes:

Since, moreover, individuals are tempted to avoid such tasks, art will tackle the most difficult and most important ones where it is able to mobilize the masses. Today it does so in film. (Benjamin 1969, p. 240)

The film has the potential to mobilize the masses, however it does not do it for the right reasons, Benjamin seems to be concluding.

However, relating and reading Bloch’s theory of escapism and colportage as a pedagogical possibility I have argued in this article that the fictional stories of TV series *can have* a potentiality of creating change. Also, as Lawler (2000) points out, the right reasons can differ, and one needs to ask the question: though which lens can escapism be regarded as a failure? One need to ask the question, whose escape, and from where? In order to make that judgement.

Bloch's theory of escapism and utopia is a political theory of action and of processes. I also read it as an understanding of how escapism and waiting are important in order to create change. Waiting and dreaming are not idle. Rather the opposite, as a possibility that is *not yet*. Daydreaming, going into a world of heroes, like Lettia and Atticus world in *Lovecraft Country*, for a few hours, can be regarded as peaceful or relaxing, as a rest, and *a not-yet time*: it is not action or can be regarded as productive life as in working or studying.

In my book (Hållander, 2020) I have explore this *not-yet* phenomenon and written about it in terms of the pedagogical possibility of *arrested time*. The pedagogical possibility, I argue drawing on Giorgio Agamben, is always related to the things that do not pass into actuality, to the not-doing and not-yet, to the impossibility. The arrested time can be understood in terms of a position of beginnings. I quote Agamben from the book *Potentialities*: “It [the existence of potentiality] is potentiality that is not simply the potential to do this or that thing but potential to not-do, potential not to pass into actuality” (Agamben 1999, s. 180). In relation to this I have argued that the pedagogical possibility is always the start and the beginning of change. It is not-yet, but also, always aspiring, which can as well as might not pass in to actuality. I write:

This idea of the arrested time, or of the rupture that can interrupt telos, may be of value for teaching that wants to use the shared memory, of two sides, as a starting point. The arrested time makes it possible to interrupt, revise, and perhaps thereby overthrow the predetermined. There are pedagogical possibilities in the arrested time. (Hållander, 2020, p. 100)

Following this, I relate the escapist act as a pedagogical possibility, as a way of enacting new beginnings. It is not a failure for the masses, but a potentiality. With Bloch’s words, it is an immature, but still honest, substitution for a revolution.

## Conclusion: Escapism as a Pedagogical Possibility

“Burn under your wrath/with the authority’s news/Do not dampen your pain/over life/stolen from us/Don’t comfort your sorrow/over the world/being raped/in front of our eyes/Fire under your wrath” (Sjöstrand 1979, p. 48. My translation.)^4^

What are the pedagogical possibilities of escapism and TV series? In this article, drawing on Bloch and Benjamin, I have explored this question through a discussion of the consumption of cinema and of TV series as forms of escapism and daydreaming. Rather than seeing them as something simple, I have sought to depict how they can be thought of in another way, as forms of seeds for a new social order and utopia and thus as having pedagogical possibility. To imagine something different, can be a beginning for change (Bloch 1986). One can use cinema, as the Swedish poet Ingrid Sjöstrand quoted above wrote, to “Burn under your wrath.” To do this, cinema can be this event that lights that fire, that starts thoughts and reflections of how the world is situated and can be. The pedagogical possibility that I am arguing for here is of course not something that is present at all times: I see the problems and challenges with students (and myself) being drawn into TV series instead of studying, and therefore not a desirable part of teaching. But in order to understand the potentiality of transformation, one also needs to understand the processes that precedes those events of change. Therefore, I argue that escapism can be a form of utopian dreaming that could be the seed of changing things. In this I have regarded escapism as moments of pedagogical possibilities, as moments of possible transformations. As I argued earlier, the pedagogical possibility can consist of an arrested time where action becomes possible (Hållander, 2020). The question therefore becomes, when do we grasp that possibility? In teaching one can recognize those events as not opposed to studying, but a part of them, by saying: let us escape for one moment—and after that: Let us move on and study!

## Footnotes

1 For example, when Norwegian youth series *SKAM* was shown, the characters used Instagram to extend and portray the daily lives beyond the TV series. In these accounts, one could follow Sana Bakkoush, who wrote, “Dear haters, I have so much more for you to be mad about. Just be patient”, Noora Sætre, and see her buy toilet paper and Vilde Hellerud, who posted putting on lipstick from a particular brand. Through that they, the TV show, also blur the line between reality and fiction, advertising and politics. As a viewer, one could be distracted by it, one could be drawn into it, participate through social media, wanting and longing for the life that was being portrayed, and they, the producers of the TV series, could use that and respond to it.

2 The time aspect in relation to escapism is also interesting as, within the form of escapism that I am interested in here, time seems to ‘fly away’: 1 h is nothing, 3 h hardly noticed. The term *Binge watching* covers this phenomenon well; to watch a whole season in one night is nothing (Matrix 2014). Having worked in various industries, restaurants and agriculture where I did a lot of repetitive movements, I have experienced how escapism had this function of making time fly. It made the day pass: I could do my things, my work, without thinking about what my hands were doing. Instead, and at the same time, my mind could go wildly off into something far away, or nearby, but something different than what I was doing.

3 I have also developed this idea of the fairy tales of netflix as colportage in an short essay called “Aldrig mer det vardagliga” published at Arena Essä (2021).

4 Original: “Elda under din vrede/med maktens nyheter/Dämpa inte din smärta/över livet/som stjäls ifrån oss/Trösta/inte din sorg/över världen/som våldtas/inför våra ögon/Elda under din vrede”.

## References

[CR1] Agamben G (1999). Potentialities: Collected Essays in Philosophy.

[CR2] Arendt H (1998). The Human Condition.

[CR3] Benjamin W (1969). Illuminations: Essays and Reflections.

[CR4] Bloch E (1986). The Principle of Hope.

[CR5] Cook J (2018). Imagined Futures: Hope, Risk and Uncertainty.

[CR6] Freire P, Freire AMA (2004). Pedagogy of Hope: Reliving Pedagogy of the Oppressed.

[CR8] Hållander, M. 2020. *The Pedagogical Possibilities of Witnessing and Testimonies. Through the Lens of Agamben. *Palgrave Pivot. 10.1007/978-3-030-55525-2.

[CR20] Hållander, M. 2021. Aldrig mer det vardagliga, Arena Essä, https://www.dagensarena.se/essa/aldrig-mer-det-vardagliga/

[CR7] Hedman, D. 2017. Den Tidiga Svenska Kriminalberättelsen. *Litteraturbanken*. https://litteraturbanken.se/presentationer/specialomraden/DenTidigaSvenskaKriminalberattelsen.html.

[CR10] Lawler S, Munt IS (2000). Escape and Escapism: Representing Working-class women. Cultural Studies and the Working Class: Subject to Change.

[CR11] Lewis TE (2013). On Study: Giorgio Agamben and Educational Potentiality.

[CR9] Masschelein Jan, Simons Maarten (2013). In Defence of the School. A Public Issue.

[CR12] Matrix S (2014). The Netflix Effect: Teens, Binge Watching, and On-Demand Digital Media Trends. Jeunesse: Young People, Texts, Cultures.

[CR13] Papastephanou M (2016). Needing ‘Tomorrow as Fish Need Water’: Dystopia, Utopia, and Freire’s Pedagogy. Interchange.

[CR14] Papastephanou M (2018). Before, Now and After. Educational Philosophy and Theory.

[CR15] Rytzler, J. 2017. *Teaching as Attention Formation: A Relational Approach to Teaching and Attention*. Mälardalen University. http://mdh.diva-portal.org/smash/record.jsf?pid=diva2:1066806.

[CR16] Sjöstrand I (1979). Det blåser en sol: [Systror].

[CR17] Suissa J (2001). Anarchism, Utopias and Philosophy of Education. Journal of Philosophy of Education.

[CR18] *Svenska akademiens ordlista över svenska språket*. 2006. Svenska Akademien. http://www.svenskaakademien.se/ordlista.

